# An interspecific foraging association with polar bears increases foraging opportunities for avian predators in a declining Arctic seabird colony

**DOI:** 10.1002/ece3.11012

**Published:** 2024-03-11

**Authors:** Andrew F. Barnas, Cassandra A. B. Simone, Erica A. Geldart, Oliver P. Love, Patrick M. Jagielski, H. Grant Gilchrist, Evan S. Richardson, Cody J. Dey, Christina A. D. Semeniuk

**Affiliations:** ^1^ Department of Integrative Biology University of Windsor Windsor Ontario Canada; ^2^ School of Environmental Studies University of Victoria Victoria British Columbia Canada; ^3^ National Wildlife Research Centre, Science and Technology Branch Environment and Climate Change Canada Ottawa Ontario Canada; ^4^ Science and Technology Branch Environment and Climate Change Canada Ottawa Ontario Canada

**Keywords:** animal behaviour, drone, eider, foraging ecology, parasitism, species interaction

## Abstract

Interspecific foraging associations (IFAs) are biological interactions where two or more species forage in association with each other. Climate‐induced reductions in Arctic sea ice have increased polar bear (*Ursus maritimus*) foraging in seabird colonies, which creates foraging opportunities for avian predators. We used drone video of bears foraging within a common eider (*Somateria mollissima*) colony on East Bay Island (Nunavut, Canada) in 2017 to investigate herring gull (*Larus argentatus*) foraging in association with bears. We recorded nest visitation by gulls following *n* = 193 eider flushing events from nests during incubation. The probability of gulls visiting eider nests increased with higher number of gulls present (β = 0.14 ± 0.03 [SE], *p* < .001) and for nests previously visited by a bear (β = 1.14 ± 0.49 [SE], *p* < .02). In our model examining the probability of gulls consuming eggs from nests, we failed to detect statistically significant effects for the number of gulls present (β = 0.09 ± 0.05 [SE], *p* < .07) or for nests previously visited by a bear (β = −0.92 ± 0.71 [SE], *p* < .19). Gulls preferred to visit nests behind bears (χ^2^ = 18, df = 1, *p* < .0001), indicating gulls are risk averse in the presence of polar bears. Our study provides novel insights on an Arctic IFA, and we present evidence that gulls capitalize on nests made available due to disturbance associated with foraging bears, as eiders disturbed off their nest allow gulls easier access to eggs. We suggest the IFA between gulls and polar bears is parasitic, as gulls are consuming terrestrial resources which would have eventually been consumed by bears. This finding has implications for estimating the energetic contribution of bird eggs to polar bear summer diets in that the total number of available clutches to consume may be reduced due to avian predators.

## Graphical abstract

Interspecific foraging associations (IFAs) are biological interactions where two or more species forage in association with each other. Climate‐induced reductions in Arctic sea ice have increased polar bear (*Ursus maritimus*) foraging in seabird colonies, which creates foraging opportunities for avian predators. Our study provides novel insights on an IFA between Herring gulls (*Larus argentatus*) and polar bears, and we present evidence that gulls capitalize on seabird nests made available due to disturbance associated with foraging bears.

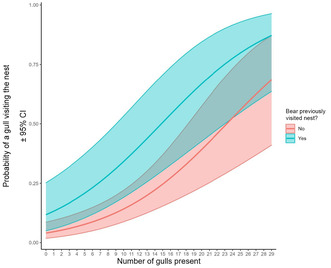

## 1 INTRODUCTION

Interspecific foraging associations (IFAs) are biological interactions where two or more species forage in association with each other (Haynes et al., 2011; Sridhar et al., 2009; Stensland et al., 2003; Thornton et al., 2018). These relationships can take many different forms including parasitism (e.g., Australian pelicans *Pelecanus conspicillatus* stealing food from pied cormorants *Phalacrocorax varius* (Love & Semeniuk, 2002)), mutualism/collaboration (e.g., coyotes *Canis latrans* and American badgers *Taxidea taxus* increasing each other's foraging efficiency by scaring prey into the other species' respective habitat (Minta et al., 1992)), or commensalism (e.g., Greater anis *Crotophaga major* foraging on terrestrial arthropods disturbed by passing freshwater fishes (Ubaid, 2011)). IFAs can involve complex social relationships, with evolved specialized roles for participating members (Bshary et al., 2006; Sampaio et al., 2020), but can also exist as transient short‐term interactions involving learned responses by individuals (Diamant & Shpigel, 1985; Silveira et al., 1997). Many IFAs involve a relatively simple interaction whereby a leader species (also referred to as “nuclear species” e.g., Somaweera & Somaweera, 2021) is primarily responsible for securing, or increasing the availability of food resources, and a follower species benefits from the actions of the leader (Sridhar et al., 2009; Strand, 1988; Thornton et al., 2018). Followers are typically smaller generalists (Sridhar et al., 2009) and can benefit from correctly associating foraging leaders with increased prey availability, thereby reducing their own search time and energy expenditure in securing food (Brockmann & Barnard, 1979; Stahler et al., 2002).

IFAs have been well described within several different taxonomic groups including between mammals (Minta et al., 1992; Thornton et al., 2018), birds (Love & Semeniuk, 2002; Sridhar et al., 2009), and fish (Strand, 1988), but can also involve relationships among different taxonomic groups (Gatti et al., 2021; Ridoux, 1987; Sakamoto et al., 2009; Somaweera & Somaweera, 2021). A frequently reported association in terrestrial systems is that between a leader mammal and a follower bird species (Booth‐Binczik et al., 2004; Fontaine, 1980; Silveira et al., 1997; Stahler et al., 2002). IFAs involving follower birds and leader mammals may be likely to arise as birds are highly mobile, and their overhead aerial view with acute vision (Opermanis, 2004) allows easier identification of foraging mammals and resulting prey. Conversely, larger mammals may be more likely to secure food items that would otherwise be inaccessible to smaller avian predator species (Stahler et al., 2002).

One such IFA between mammals and birds that has been suggested in the literature, but lacks quantitative examination, is that of the association between terrestrial foraging polar bears (*Ursus maritimus*) and avian predators (Gaston & Elliott, 2013; Iverson et al., 2014; Jagielski, Dey, Gilchrist, Richardson, & Semeniuk, 2021). Climate‐induced reductions in spring sea ice are forcing bears ashore earlier in the year in many populations (Derocher et al., 2004; Lunn et al., 2016; Regehr et al., 2007), which has led to increased foraging on several nesting bird species (Barnas, Darby, et al., 2022; Barnas, Iles, et al., 2020; Iverson et al., 2014; Jagielski, Dey, Gilchrist, Richardson, Love, & Semeniuk, 2021; Jagielski, Dey, Gilchrist, Richardson, & Semeniuk, 2021; Prop et al., 2013; Rockwell & Gormezano, 2009; Smith et al., 2010). As bears move through nesting bird colonies, incubating parents can be disturbed off their nests (Barnas, Geldart, et al., 2022; Gaston & Elliott, 2013; Jagielski, Dey, Gilchrist, Richardson, Love, & Semeniuk, 2021; Simone et al., 2022), but see Barnas, Darby, et al. (2022), leading to an increase in unguarded eggs that are more easily accessible to avian predators (Harvey, 1971; Inglis, 1977; Prop et al., 1985). Disturbance foraging by avian predators is well documented during researcher activities in Arctic bird colonies (Åhlund & Götmark, 1989; Bêty & Gauthier, 2001; Götmark, 1992; Götmark & Åhlund, 1984), and increased bear presence in Arctic bird colonies will likely lead to greater disturbances, thus creating more foraging opportunities for avian predators. This indirect effect of climate change is particularly concerning as changes to predator foraging strategies may have a disproportionately high impact on the relatively simple food webs of low‐productivity Arctic ecosystems (Krebs et al., 2003; Seyer et al., 2020).

Avian predators act as scavengers of polar bears on sea ice by making use of seal carcasses and other carrion left by bears (Derocher, 2012; Secretariat, 2015; Spencer et al., 2016), and have been observed following bears in nesting bird colonies (Gaston & Elliott, 2013; Madsen et al., 2019). However, beyond increasing the availability of nests for avian predators, the actual characteristics of the IFA and factors impacting foraging efficiency for avian predators are relatively understudied. For example, there remain knowledge gaps of the characteristics of bear foraging that make certain nests more or less likely to be visited by avian predators (given the attendant prey‐parent has flushed). Foraging in the presence of polar bears is also potentially risky for avian predators, as bears have been observed to capture adult birds while on land (Gormezano et al., 2017; Iles et al., 2013). While foraging avian predators are more mobile than incubating parents or seasonally flightless adults, there remains a predation risk by bears to these follower species (Stempniewicz, 2006). Behaviours of incubating parent birds can also influence predation risk, as increased parental activity at nests may serve as visual cues for both avian predators and bears (Barnas, Geldart, et al., 2022; Simone et al., 2022).

The objectives of this exploratory study are to investigate the interspecific foraging association between avian predators and foraging polar bears in a nesting colony of common eiders (*Somateria mollissima*). We examine several aspects of the proposed IFA. Eiders have extremely high nest attendance behaviours, and egg predation by avian predators typically occurs when eider parents are off nest (Criscuolo et al., 2000). Bear foraging, which disturbs eiders off their nest, is likely to create foraging opportunities for herring gulls. While bear foraging on East Bay Island is known to result in near complete nest failure for eiders (Barnas, Geldart, et al., 2022; Jagielski, Dey, Gilchrist, Richardson, & Semeniuk, 2021), the role of an IFA is unclear. We make use of aerial drone videography collected during polar bear foraging in an eider breeding colony, and the foraging behaviour of an avian predator, herring gulls (*Larus argentatus*) during these events. Specifically, we examine the probability of gulls visiting eider nests and the probability of gulls consuming eggs from an eider nest following disturbance by a polar bear. We examine the influence of two factors on these probabilities: (1) the number of gulls present at the time of flush, and (2) whether or not a bear had previously visited the nest (after an eider flushed). We predicted both probabilities (nest visitation and egg consumption) would increase with a higher number of gulls present as chances of at least one individual gull detecting a nest should increase as gull numbers increase. We similarly predicted increased probabilities of nest visitation and egg consumption from eider nests that were previously visited by bears, as the presence of a bear at the nest should provide visual cues on nest locations for gulls. Lastly, we predicted that gulls would be more likely to visit eider nests from behind as the bears moved away from the area as opposed to in front of bears moving through the colony, as this would provide scavenging opportunities and reduce mortality risk for gulls following polar bears.

## 2 METHODS

### 2.1 Study site

**FIGURE 1:**
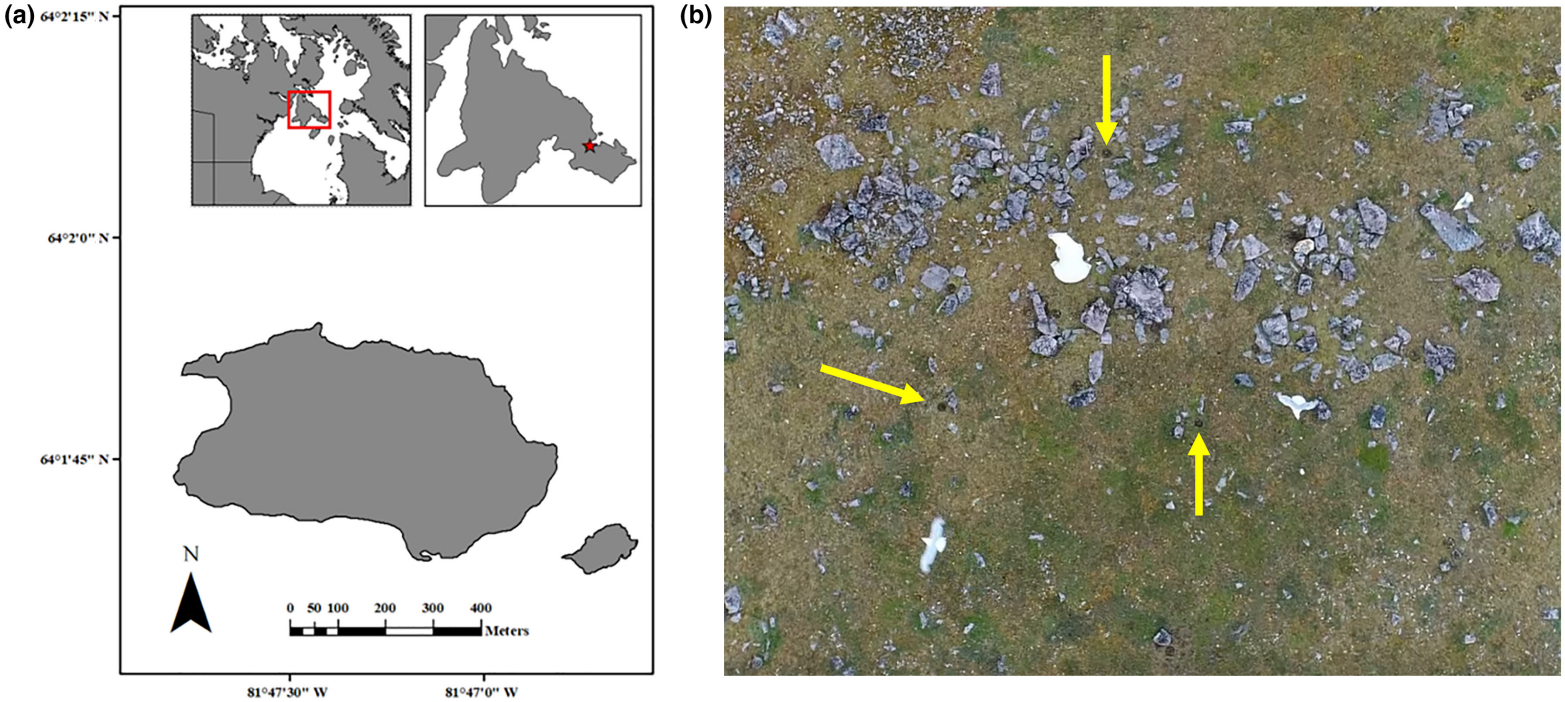
(a) Location of East Bay Island, Nunavut (Canada) in northern Hudson Bay. Red star represents location of the East Bay Island, within the East Bay of Southampton Island. Canadian Provinces and Territories map layers provided by ESRI online, accessed September 1st, 2020. (b) Example drone footage captured on July 15th 2017 at 30 m AGL of herring gulls (*Larus argentatus*) foraging alongside a polar bear (*Ursus maritimus*) in a common eider (*Somateria mollissima*) breeding colony. Yellow arrows point to examples of empty eider nest bowls.

Data were collected in the summer of 2017 on East Bay (Mitivik) Island, within the East Bay (Qaqsauqtuuq) Bird Sanctuary of Southampton Island, Nunavut, Canada (Figure 1). East Bay Island is a relatively small (approximately 0.24 km^2^) island with flat topography (total elevation change, approximately 8 m). This study site has historically hosted the largest known eider colony in the Canadian Arctic, with up to 8000 breeding pairs (Jean‐Gagnon et al., 2018), however recent estimates are much lower with approximately 1500–1700 breeding pairs in 2017 (Jagielski, Dey, Gilchrist, Richardson, & Semeniuk, 2021). Although a small number of solitarily‐breeding king eiders (*Somateria spectabilis*) breed on the island (mainly along the coastline, OPL/HGG pers. obs.), we assume that the observed individuals are common eiders as females of each species are difficult to distinguish from drone video. This area is seasonally ice‐free and has long served as an important summering ground for the Foxe Basin subpopulation of polar bears (Sahanatien et al., 2015; Stapleton et al., 2016), but bears have now begun arriving on East Bay Island more frequently and earlier during the eider incubation period in recent years resulting in reproductive failure for eiders (Iverson et al., 2014; Jagielski, Dey, Gilchrist, Richardson, & Semeniuk, 2021). The main avian predators of eiders on East Bay Island are herring gulls of which the island hosts approximately 30 nesting pairs annually (Allard, 2006), although other avian predators such as parasitic jaegers (*Stercorarius parasiticus*) are known to consume unattended eider eggs on the island (Bottitta, 1999). We noted zero observations of jaegers during the study. Similarly, Arctic foxes (*Vulpes lagopus*) were not observed during the study period.

### 2.2 Drone video collection and review

To observe interactions between gulls, eiders and bears, we examined video footage collected by multi‐rotor drones (Chapman, 2014) between July 10th and 20th in 2017 on East Bay Island. To the best of our knowledge, there are no studies on herring gull responses to drone surveys; however, herring gulls have been shown to steal eggs from common murres (*Uria aalge*) as murres flushed in response to drones (Brisson‐Curadeau et al., 2017). Quadcopter‐style drones flown at 30 m above ground level (the minimum height of drones flown in our study) do not illicit a physiological (e.g., heart rate) response in nesting eiders (Geldart et al., 2022). The only studies examining behavioural responses of nesting eiders or polar bears to drones use fixed‐wing models, and neither species showed adverse behavioural reactions to these aircraft (Barnas et al., 2018; Ellis‐Felege et al., 2021). When polar bears were seen foraging on East Bay Island, a drone was deployed to film a single bear as it moved through the colony searching for eider nests. We did not review any footage which contained multiple bears in frame. This footage was originally collected to estimate the energetics of bear foraging on eider eggs, and the authors recorded 31 foraging bouts from 20 individual bears (Jagielski, Dey, Gilchrist, Richardson, Love, & Semeniuk, 2021; Jagielski, Dey, Gilchrist, Richardson, & Semeniuk, 2021). This dataset was previously analyzed to examine the spatial effects of bear foraging on eider flush behaviour (Barnas, Geldart, et al., 2022); however, for this current research we reviewed a subset of the original footage optimized for collecting behavioural observations of eider flush events and gull foraging. We only reviewed footage with a nadir (straight down) view, collected at approximately 30–55 m Above Ground Level (AGL). We chose this subset of the drone footage to maintain a consistent field of view when scoring eider, bear, and gull behaviours. As a result, we reviewed 166.3 min of drone video across 15 drone flights. The field of view for these videos was estimated using 10 random screenshots of video and measuring the length and width of frames alongside a georeferenced map of East Bay Island. The mean ± SD area of video frames was 1023 ± 195 m^2^, suggesting a reasonably consistent field of view. For a detailed description on the original collection of the drone footage and processing see Jagielski, Dey, Gilchrist, Richardson, Love, and Semeniuk (2021); Jagielski, Dey, Gilchrist, Richardson, and Semeniuk (2021), and Barnas, Geldart, et al. (2022) along with the associated drone reporting protocol (Barnas, Chabot, et al., 2020).

Two observers (AB and CABS) reviewed video footage for flushing eider hens, along with bear and gull behaviour following eider flushes using Windows Film & TV application v.10.200022.11011 (Microsoft Corporation, Washington, USA). In some cases, eiders flushed from their nest, returned to their nest, and then flushed again during the same bear foraging bout. In other cases, the same eider was observed to flush from a bear on the following day (determined using natural landmarks to re‐identify nests). In both of these scenarios, we only used the first recorded flush event for an individual. When an eider flushed, we recorded the number of gulls observed in the paused video frame. Following each eider's flush, we recorded if the bear or any gulls visited the nest and whether either predator consumed eider nest contents (hereafter referred to as “eggs,” although ducklings may have been present at this time, see Simone et al., 2022). We defined nest visits for bears and gulls as behaviours where the predator approached the focal nest and appeared to touch the eider nest bowl or contents.

Evidence of egg consumption by bears and gulls from drone footage included observing the predator consuming eggs, extended time periods spent digging through nest materials, or reduction in the number of eggs observed within the eider nest before versus after the predator visited. Note it was not always clear whether nests were entirely emptied by bears versus those which had materials still available to gulls, and we did not attempt to record information on partial predations. We only considered predation (or a lack of predation) for nests during the time they were visible in the drone's field of view. As a result, we potentially missed predation events after the drone moved away, but here we consider the immediate impacts of bear presence on gull foraging, rather than impacts due to potentially prolonged eider absences from the nest.

We recorded descriptive statistics for the number of gulls visiting each nest, as well as the time (s) it took for individual gulls to visit the nest following the initial eider flush. For example, if an eider left the nest at 10:15 (MM:SS) in the video, and the gull visited at 10:45 (MM:SS), this would be 30 s. To describe foraging behaviour of gulls in relation to the position of bears, we recorded the manner of gull nest visitation and egg consumption relative to the forward‐facing angle of the bear. We recorded how gulls visited (and consumed eggs from) nests if they visited “behind” or “in front” of the forward‐facing direction of the bear. We did not record information on the specific behaviours of how gulls foraged at the nest, for example pecking eggs at the nest or stealing whole eggs away.

### 2.3 Statistical analysis

To estimate the probability of a gull visiting an eider nest where the female eider flushed during polar bear foraging events, we constructed a logistic regression model with a logit link function. We included fixed effects for the number of gulls observed in‐frame when the eider initially flushed (integer, range 0–29), whether or not a bear had visited the eider nest (categorical, levels “Yes” and “No”). Next, we examined the probability of gulls consuming eggs from eider nests by constructing an additional logistic regression model for gulls that had visited nests (i.e., we used a subset of the data where gulls had visited eider nests). We included the same fixed effects as the first model. For both models we evaluated fit based on likelihood ratio tests, which compares the deviance of our candidate models to an intercept‐only (null) model, allowing us to determine if a more complex model fits better than a reduced intercept‐only model. We made model predictions on the response (probability) scale to visually represent model estimates.

We did not include measures of flush initiation distances by eiders, or other components of distance to nests in these analyses, as we have previously considered these in Barnas, Geldart, et al. (2022). For both models, we tested the inclusion of a random intercept for date of observation, as this would partially account for variation in gull responses due to foraging by the same bears or by the decreasing availability of nests throughout the nesting season. For both the gull visit model and the egg consumption model, likelihood ratio tests showed including these random effects did not significantly change model fit (respectively χ^2^ = 1.6^−08^, df = 1, *p* < .99, and χ^2^ = 6.0^−09^, df = 1, *p* < .99). Therefore, we considered only fixed effect models moving forward.

We performed chi‐square goodness‐of‐fit tests for (1) the number of nests visited only by gulls, only by bears, or both, (2) the number of gulls observed visiting eider nests either behind or in front of polar bears, and (3) the number of gulls found consuming eggs behind or in front of polar bears (given that the gull was already at the nest). These tests were performed to determine if a greater number of nests were visited or eaten by gulls in each category than expected by random chance. For all statistical tests, we used α = 0.05 as a cut‐off for statistical significance. All data management and manipulations were done using RStudio v3.6.2 (R Core Team, 2017) using package *dplyr* for general data manipulation (Wickham et al., 2015), *lubridate* for datetime calculations (Grolemund & Wickham, 2011), *lmtest* for computing likelihood ratio tests (Zeileis & Hothorn, 2002), *ggplot2* for data visualization (Wickham, 2016), and *ggeffects* for model predictions (Lüdecke, 2018). We report all model coefficients ± standard errors (SE).

## 3 RESULTS

We recorded foraging behaviours of polar bears and gulls during flush events from 193 eider nests. The distribution of flushes observed on each day was: July 11 (*n* = 61), July 15 (*n* = 97), July 16 (*n* = 34), and July 19 (*n* = 1). No flushes were observed from drone video on July 10 or 20; videos from July 10 did not meet video quality requirements, and few nests remained on East Bay Island by July 20. Polar bears were observed to visit 40 nests and consume eggs from 33 of these 40 nests (7 were visited but not consumed by bears). We recorded visitations by 50 gulls to 29 eider nests and eggs were consumed from 10 of these 29 nests (19 were visited but not consumed by gulls). A total of 13 nests were visited by both bears and gulls, but eggs were only consumed by both predators at 4 of these nests (Table 1). Mean visitation time (±SD) of gulls to nests was 157.6 ± 100.2 s (*n* = 50 gull visits due to multiple gulls visiting eider nests, range 19–396 s, Figure S1). Based on these calculations, the majority of flushed nests were observed by the drone for approximately 2.5 min (Figure S1). The mean number of gulls present when an eider left its nest was 5.9 ± 7.1 (*n* = 193 eider flushes, range: 0–29 gulls, Figure S2); similarly, for nests that were visited by gulls we found a mean of 1.7 ± 1.4 gull visiting nests (*n* = 29 nests visited by gulls, range: 1–7 gulls visiting each eider nest, Figure S3).

**TABLE 1:** Percentage of common eider (*Somateria mollissima*) nests visited and nest contents consumed by polar bears (*Ursus maritimus*) and herring gulls (*Larus argentatus*), or both.

Predator species	% of nests visited^a^	% of nests with contents consumed^b^
Polar bears	20.7% (40/193)	82.5% (33/40)
Herring gulls	15.0% (29/193)	34.5% (10/29)
Both	6.7% (13/193)	30.8% (4/13)

*Note*: Data obtained from video collected by drone on East Bay Island, Nunavut, Canada.

^a^ Number of nests visited/total number of flushes.

^b^ Number of nests consumed/number of nests visited.

**FIGURE 2:**
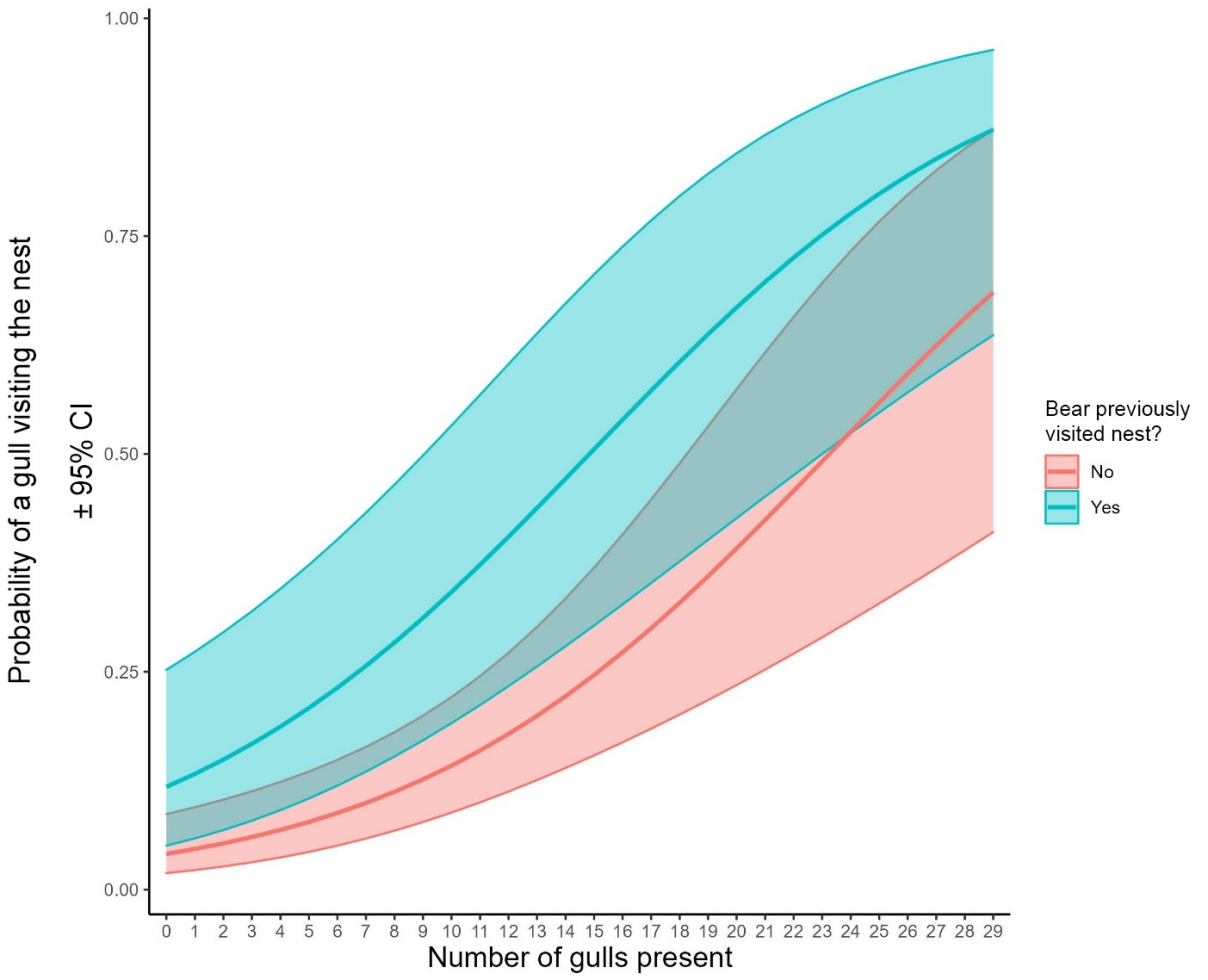
Logistic regression model predictions (±95% CI) for the probability of herring gulls (*Larus argentatus*) visiting common eider (*Somateria mollissima*) nests during polar bear (*Ursus maritimus*) foraging as a function of the number of gulls present and whether or not a bear had previously visited the nest. Model fit using *n* = 193 observations of eider flushes.

**FIGURE 3:**
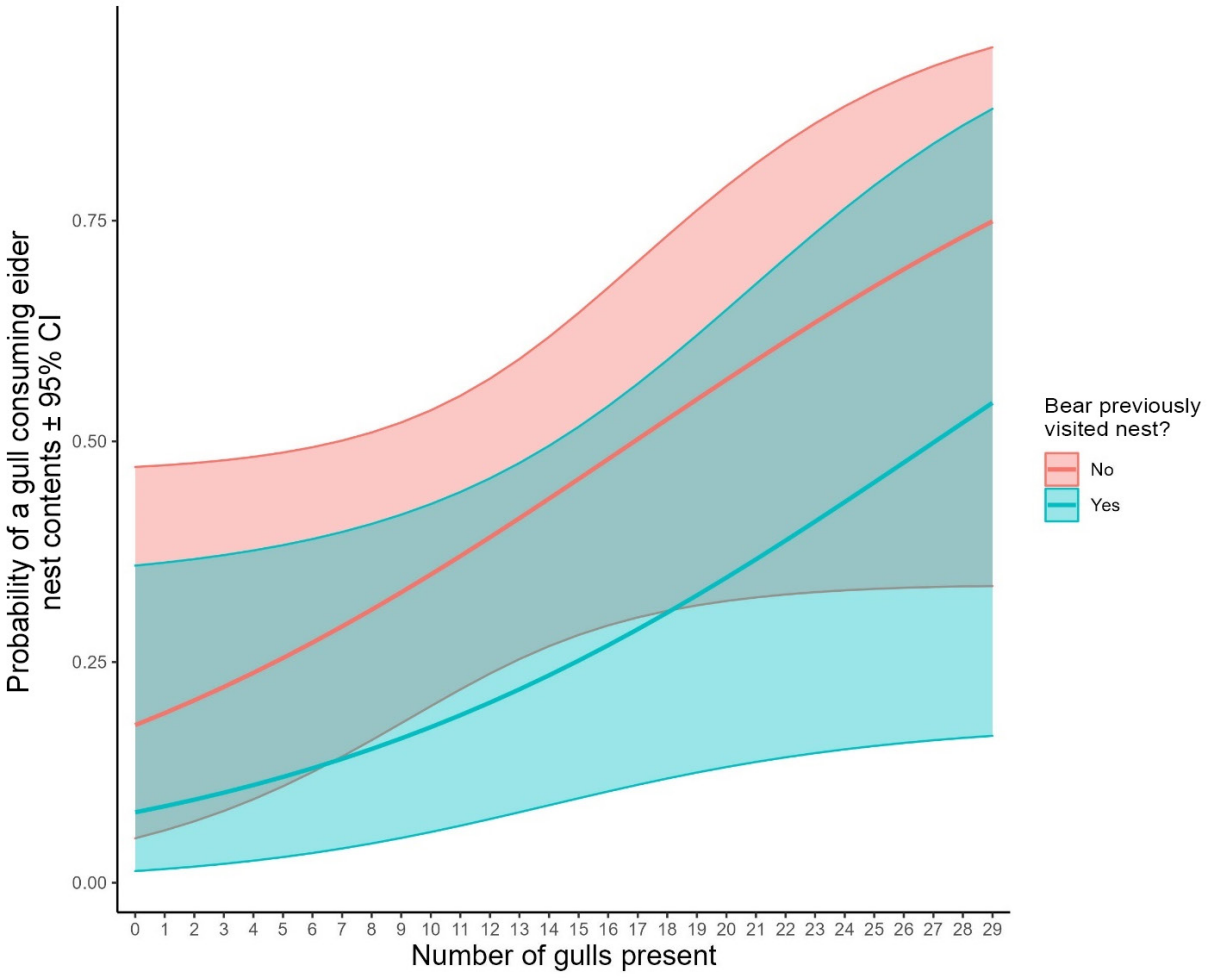
Logistic regression model predictions (±95% CI) for the probability of herring gulls (*Larus argentatus*) at common eider (*Somateria mollissima*) nests consuming eggs during polar bear (*Ursus maritimus*) foraging as a function of the number of gulls present and whether or not a bear had previously visited the nest. Model fit using *n* = 50 observations of gulls at eider nests.

We found the probability of gulls visiting eider nests was higher with increasing number of gulls present at flush (β = 0.14 ± 0.03, *p* < .001) and for nests that had previously been visited by a polar bear (β = 1.14 ± 0.49, *p* < .02) (Table 2, Figure 2). In our model examining the probability of gulls consuming eggs from eider nests, we failed to detect statistically significant effects for the number of gulls present (β = 0.09 ± 0.05, *p* < .07), or whether or not a bear had previously visited the nest (β = −0.92 ± 0.71, *p* < .19). Concordantly, based on likelihood ratio tests the model for gull visitation fit better when compared to an intercept‐only model (χ^2^ = 32.1, df = 3, *p* < .001), but the model for gull egg consumption did not (χ^2^ = 4.90, df = 3, *p* < .09) (Table 2, Figure 3).

**TABLE 2:** Fixed effect estimates from logistic regression models examining probability of herring gull (*Larus argentatus*) visitation and probability of gulls consuming eggs from common eider (*Somateria mollissima*) nests during polar bear (*Ursus mariimus)* foraging events.

Model	Intercept	Number of gulls	Did a bear visit?^a^
Gull visitation	**−3.15 ± 0.41**	**0.14 ± 0.03**	**1.14 ± 0.49**
Egg Consumption	**−1.53 ± 0.72**	0.09 ± 0.05	−0.92 ± 0.71

*Note*: Gull visitation model based on *n* = 193 observations of eider nest flushes and egg consumption model based on *n* = 50 observations of gulls which visited eider nests. Bold values denote statistical significance at α = 0.05.

^a^ Reference category = Nests that were not previously visited by bears.

**FIGURE 4:**
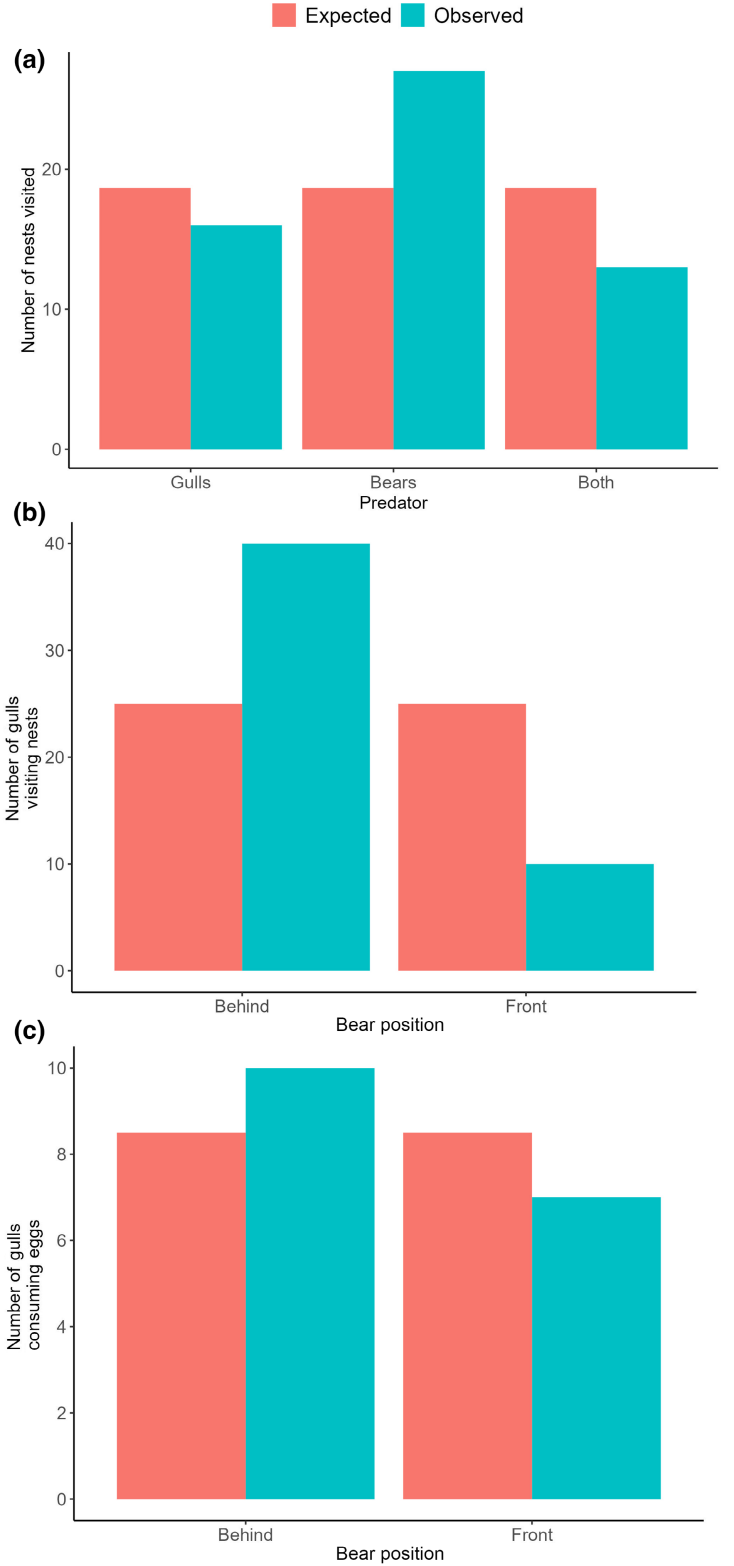
Expected and observed number of (a) common eider (*Somateria mollissima*) nests visited by herring gulls (*Larus argentatus*) only (*n* = 16), polar bears (*Ursus maritimus*) only (*n* = 27), or both (*n* = 13), (b) number of gulls visiting eider nests behind (*n* = 40) or in front (*n* = 10) of bears, (c) number of gulls consuming eggs from eider nests visited behind (*n* = 10) or in front of bears (*n* = 7).

Using chi‐square tests, we failed to find a statistically significant difference in the number of nests visited by gulls, bears, or both (χ^2^ = 5.82, df = 2, *p* < .054, Figure 4a), but when gulls visited nests, they preferred to do so behind bears as opposed to in front of bears (χ^2^ = 18, df = 1, *p* < .0001, Figure 4b). Once gulls had arrived to nests, there was no statistical difference in whether or not eggs were consumed relative to arrival behind or in front of bears (χ^2^ = 0.53, df = 1, *p* < .47, Figure 4c).

## 4 DISCUSSION

Strong effects of climate change can arise through changes in biological interactions (Parmesan, 2006; Walther et al., 2002), and Arctic systems are particularly sensitive to changing species relationships due their relatively simple food webs (Bêty et al., 2002; Seyer et al., 2020). Our study provides novel insights on an IFA in the Arctic between herring gulls and polar bears foraging in a common eider colony. We present evidence that gulls do visit nests made available due to disturbance associated with foraging bears; nests that would have been more difficult to access by gulls due to the presence of incubating eiders (Bolduc & Guillemette, 2003; Criscuolo et al., 2000). Disturbances which force eiders off nests are important as eider eggs are more at risk to herring gulls in the absence of parents. Glaucous gulls (*Larus hyperboreus*) can force female eiders off nests (Allard, 2006), but this was not observed by herring gulls during our study. Although our findings are site‐specific, the demonstration of this IFA corroborates reports in other terrestrial systems that avian predators will associate with foraging bears in nesting bird colonies (Barnas, Darby, et al., 2022; Barry, 1967; Gaston & Elliott, 2013; Iverson et al., 2014; Madsen et al., 1989; Rode et al., 2015; Secretariat, 2015). We were unable to distinguish individual gulls from each other unless multiple gulls were in frame at the same time. As a result, different foraging bouts from bears may involve the same individual gulls, and extrapolations to population‐level behaviours of gulls need be done with caution.

In IFAs involving birds and terrestrial mammals, follower bird species may rely on leader mammals for procuring prey in several ways including killing larger prey species (Stahler et al., 2002), opening carcasses (Moleón et al., 2014), or flushing small prey species due to disturbance (Booth‐Binczik et al., 2004; Fontaine, 1980; Silveira et al., 1997). Herring gulls can capitalize on polar bears to flush eiders from their nest and were more likely to visit nests previously visited by bears. This suggests bears provide a strong visual cue on eider nest location, but given the high visual acuity of herring gulls (Frings et al., 1955; Tinbergen, 1953), gulls do not necessarily require bears to signal nest locations. Gulls may also not forage immediately alongside bears since bears themselves may prove a mortality risk to adult gulls, as polar bears are capable of killing flight‐capable adult birds (Gormezano et al., 2017). Common ravens (*Corvus corax*) prefer to feed at carcasses when wolves (*Canis lupus*) are present, but are sometimes killed by the wolves at these carcasses (Stahler et al., 2002), indicating avian predators that capitalize on large‐mammal presence to obtain prey may face risks alongside rewards. This is in line with our findings that when gulls visited eider nests, they preferred to do so behind polar bears. Herring gulls are risk‐averse foragers and may perceive higher risks from direct angles of eye gaze or approach angle (Burger & Gochfeld, 1981; Goumas et al., 2019, 2020). An important caveat here is that as the bear moves through the eider colony, more nests may be available “behind” the bear than ahead (although eiders can flush up to 25 m from polar bears, Barnas, Geldart, et al., 2022). However, bears do disturb eiders “ahead” of themselves, and if eiders return to their nests once the bear has passed this would somewhat negate this caveat. Although we did not record specific foraging behaviours of gulls here, different foraging methods (e.g., pecking eggs versus stealing whole eggs and taking them elsewhere) may indicate different levels of perceived risk by gulls, which should be examined in future works. Polar bears may also have impacts on eiders at greater spatial scales than we observed with the drone (and therefore foraging opportunities for gulls), but this is beyond the scope of the current study. Furthermore, impacts of the IFA may be more pronounced earlier in the nesting season as more nests are available to be targeted, and decrease as nests become less available throughout the season.

Nest predation rates by gulls during polar bear foraging were relatively low (11 nests predated from 193 flush events), and it was also surprising to find that not all gulls which visited a nest were observed to forage at that nest. An important caveat of our study is the drone video was originally collected to follow the polar bear while it foraged in the eider colony, thus the drone's field of view often moved away before eiders were observed to return to their nest. Greater time spent off nest by eiders likely increases the risk of nest predation (Bolduc & Guillemette, 2003; Swennen et al., 1993), and previous work at East Bay Island shows eiders flush more discretely when more gulls are present, likely to avoid being noticed by gulls (Barnas, Geldart, et al., 2022). Monitoring how long eiders take to return to their nest may prove informative in explaining low observed predation by gulls, as eiders will aggressively defend their nests against gulls (Allard, 2006; Reed et al., 2007). Undoubtedly some additional nests were visited and consumed by gulls in the absence of the eider outside the drone's field of view, but this represents a logistic trade‐off between low‐altitude flights with high video resolution, and higher‐altitude flights that would observe a greater field of view but with lower video resolution. We caution interpretation of our findings of gull predation which may underrepresent the total nests consumed by gulls, in that we are examining the immediate impact of bear presence on gull foraging behaviour, rather than potential lingering effects due to prolonged eider absence from the nest. As such, our results may represent a conservative baseline estimate of gull predation on eiders during polar bear foraging.

Polar bears foraging at bird nests may sometimes result in partial predations as it seems unlikely that 100 per cent of nest contents (yolk, albumen, embryos, ducklings) would be consumed by the bear and some scraps would remain, in which case it should still be worth investigating by gulls (evidenced by 4 nests appearing to be consumed by both bears and herring gulls, see Table 1). Unconsumed nest contents by bears (also documented in Jagielski, Dey, Gilchrist, Richardson, Love, & Semeniuk, 2021) may be explained by noxious defecation on eggs by eiders (McDougall & Milne, 1978), the distracting environment of the eider colony (Simone et al., 2022), or even the messy eating habits of polar bears (see missed egg yolk in figure 3 of Barnas, Iles, et al., 2020), and all of these factors warrant future investigation. Polar bears may also be more selective for nests earlier in the eider incubation period, as suggested by Jagielski, Dey, Gilchrist, Richardson, Love, and Semeniuk (2021). For a much more detailed discussion of eider responses to polar bears and herring gulls, see Barnas, Geldart, et al. (2022).

For nests that had not been visited by bears and were assumed to contain nest contents, it is unclear why gulls would not forage at the nest given they had arrived. Eiders will sometimes defecate on their clutch of eggs to deter predation, but these defences do not seem to impact whether gulls will take eggs or not (McDougall & Milne, 1978). Risk‐averse foraging strategies in gulls during the commotion induced by bear foraging may explain why gulls did not always take time to forage at the nest, with an equal likelihood of foraging relative to bear position (Figure 4c), as they may have been unwilling to risk sticking their head in a nest in the presence of the polar bear which poses a predation risk, disrupted eiders which may return to aggressively defend nests (Allard, 2006), and conspecifics who may aggressively attempt to steal food (*Barnas personal observation*). We failed to detect a significant effect of number of gulls present or whether a bear visited a nest on whether a gull consumed eggs. It seems logical that greater number of gulls would yield a higher chance of one consuming eggs, and this may be a case of biological significance being more appropriate than statistical significance.

We found the probability of gulls visiting a nest increased with increasing gull numbers, which is unsurprising as there are simply more individuals present and the chances that at least one gull visits increases with group size. Our observations of multiple gulls visiting the same eider nest may indicate social information transmission on nest location, but other gull species do not rely on social information to locate food (Andersson et al., 1981; Racine et al., 2012). Increased numbers of gulls likely also contribute to group vigilance to some degree (Beauchamp, 2009), but this may also represent increased competition with conspecifics for a finite food resource (although we found no statistically significant effect of gull number on probability of foraging). Previous work on East Bay Island showed that eiders performed more inconspicuous flush responses during polar bear foraging when more gulls were present, suggesting eiders attempt to dampen visual cues that gulls could use to locate nests (Barnas, Geldart, et al., 2022). Increased activity by parent birds at their nests increases the chances of nest discovery by predators (Martin, Martin, et al., 2000; Martin, Scott, & Menge, 2000). While eider behaviours may reduce nest loss by polar bears, in the presence of this bear‐gull IFA, such increased behavioural activity directed at bears may instead alert and attract gulls to nests and warrants future investigation.

Whether the relationship between gulls and bears on East Bay Island is definitively commensal or parasitic remains unknown, and would require future confirmatory studies to address. Within an individual polar bear's foraging bout, it is unclear whether all the nests visited by gulls would have been visited by bears as well. This indicates the relationship may be commensal, as gulls gain access to nests at no cost to polar bears. However, on East Bay Island, polar bears are inefficient foragers on eider eggs and can be present on the island throughout the nesting season, increasing energy expenditure searching for the diminishing number of remaining eider nests (Jagielski, Dey, Gilchrist, Richardson, Love, & Semeniuk, 2021). So, at a longer time scale, the nests consumed by gulls (that were not originally visited by bears during a foraging bout) would eventually have been discovered by bears (unless the bear left the island and did not return later in the season). Therefore, it is possible the IFA between gulls and polar bears is parasitic, in that gulls are consuming terrestrial resources which would have eventually been consumed by bears. This has implications for estimating the energetic contribution of bird eggs to polar bear summer diets (Gormezano & Rockwell, 2015), in that the total number of available clutches to consume may be reduced due to avian predator presence. Although the number of nests predated by gulls in our exploratory study was quite low, our sample represents a small window of time during the eider nesting period, and gulls likely consume more nests than we observed. The nature of IFAs between bears and avian predators in other nesting colonies should be investigated, as these relationships may shift to more commensal (as opposed to parasitic) in larger nesting colonies or cliff‐nesting species where bears cannot consume all nests. Although some terrestrial mammals may use avian scavengers to locate prey for themselves (Kane & Kendall, 2017), it seems unlikely that the gull‐polar bear IFA is mutualistic whereby polar bears would use gulls to locate and procure eider nests, as polar bears can locate eider nests by searching the colony (Gormezano et al., 2017; Prop et al., 2013) or even using flushing parent birds as cues (Jagielski, Dey, Gilchrist, Richardson, Love, & Semeniuk, 2021). While characteristics of eider colonies such as colony size and distance from mainland influence the probability of bear visitation (Dey et al., 2017; Iverson et al., 2014), it would be worthwhile to investigate whether foraging gulls attract bears to eider colonies. Further, continued years of the bear‐gull IFA on East Bay Island may increase foraging efficiency for bears, gulls, or both, as a learned response.

Common eiders are an ecologically and culturally significant species (Clyde et al., 2021; Henri et al., 2018), and our study provides insights on climate‐induced changes in the predator community of eiders on East Bay Island. We demonstrated that avian predators capitalize on disturbance foraging by polar bears, and it is likely that these types of interactions are occurring in other nesting bird colonies where avian predators and polar bears co‐occur (Gaston & Elliott, 2013; Madsen et al., 2019). Simulation models predict redistribution of nesting eiders to smaller colonies in response to polar bears (Dey et al., 2017), but this has not yet been shown empirically (Dey et al., 2020). The use of drones to investigate this IFA was informative for investigating the behavioural ecology of gulls and bears, and such tools may prove useful in future investigations given they don't influence the study species of interest (Barnas et al., 2018; Ellis‐Felege et al., 2021; Geldart et al., 2022; Jagielski et al., 2022). An important trade‐off between drone survey height and video quality represents a notable pitfall of drone technology here, as we were unable to observe gull predation outside the field of view or when the drone moved on. Future work potentially using multiple drones or trail cameras (Burgar et al., 2019) to study the influence of bears on an area after bears move on would be informative. We noted no interactions between gulls and the drone used during this study, but examining the impact of drones on foraging success of gulls would be worthwhile. Investigations into avian predator‐polar bear IFAs should continue to examine biological factors (e.g., nest availability) and behavioural mechanisms in prey (e.g., distraction behaviours) that could influence follower‐species foraging.

## AUTHOR CONTRIBUTIONS

**Andrew Barnas:** Conceptualization (equal); data curation (equal); formal analysis (equal); investigation (equal); methodology (equal); resources (equal); software (equal); writing – original draft (equal); writing – review and editing (equal). **Cassandra Simone:** Conceptualization (equal); data curation (equal); formal analysis (equal); investigation (equal); methodology (equal); writing – original draft (equal); writing – review and editing (equal). **Erica Geldart:** Conceptualization (equal); data curation (equal); investigation (equal); methodology (equal); writing – original draft (equal); writing – review and editing (equal). **Oliver P. Love:** Conceptualization (equal); data curation (equal); formal analysis (equal); funding acquisition (equal); investigation (equal); methodology (equal); project administration (equal); supervision (equal); writing – original draft (equal); writing – review and editing (equal). **Patrick Jagielski:** Data curation (equal); formal analysis (equal); investigation (equal); methodology (equal); software (equal); writing – original draft (equal); writing – review and editing (equal). **Grant Gilchrist:** Conceptualization (equal); data curation (equal); formal analysis (equal); funding acquisition (equal); investigation (equal); methodology (equal); project administration (equal); supervision (equal); writing – original draft (equal); writing – review and editing (equal). **Evan Richardson:** Conceptualization (equal); data curation (equal); formal analysis (equal); funding acquisition (equal); investigation (equal); methodology (equal); project administration (equal); writing – original draft (equal); writing – review and editing (equal). **Cody Dey:** Conceptualization (equal); data curation (equal); formal analysis (equal); funding acquisition (equal); investigation (equal); methodology (equal); project administration (equal); writing – original draft (equal); writing – review and editing (equal). **Christina Semeniuk:** Conceptualization (equal); data curation (equal); formal analysis (equal); funding acquisition (equal); investigation (equal); methodology (equal); project administration (equal); writing – original draft (equal); writing – review and editing (equal).

## CONFLICT OF INTEREST STATEMENT

No conflict of interest exists.

## Supporting information


Data S1.

Data S2.

## Data Availability

Data used for analysis is included as supplementary materials.
