# Supplementary material for: An interspecific foraging association with polar bears increases foraging opportunities for avian predators in a declining Arctic seabird colony
Source: Ecol Evol. 2024 Mar 11;14(3):e11012. doi: 10.1002/ece3.11012 (PMC10926061; doi:10.1002/ece3.11012)
Supplement: Supplementary file 1 — Data S1. [file ECE3-14-e11012-s001.docx]

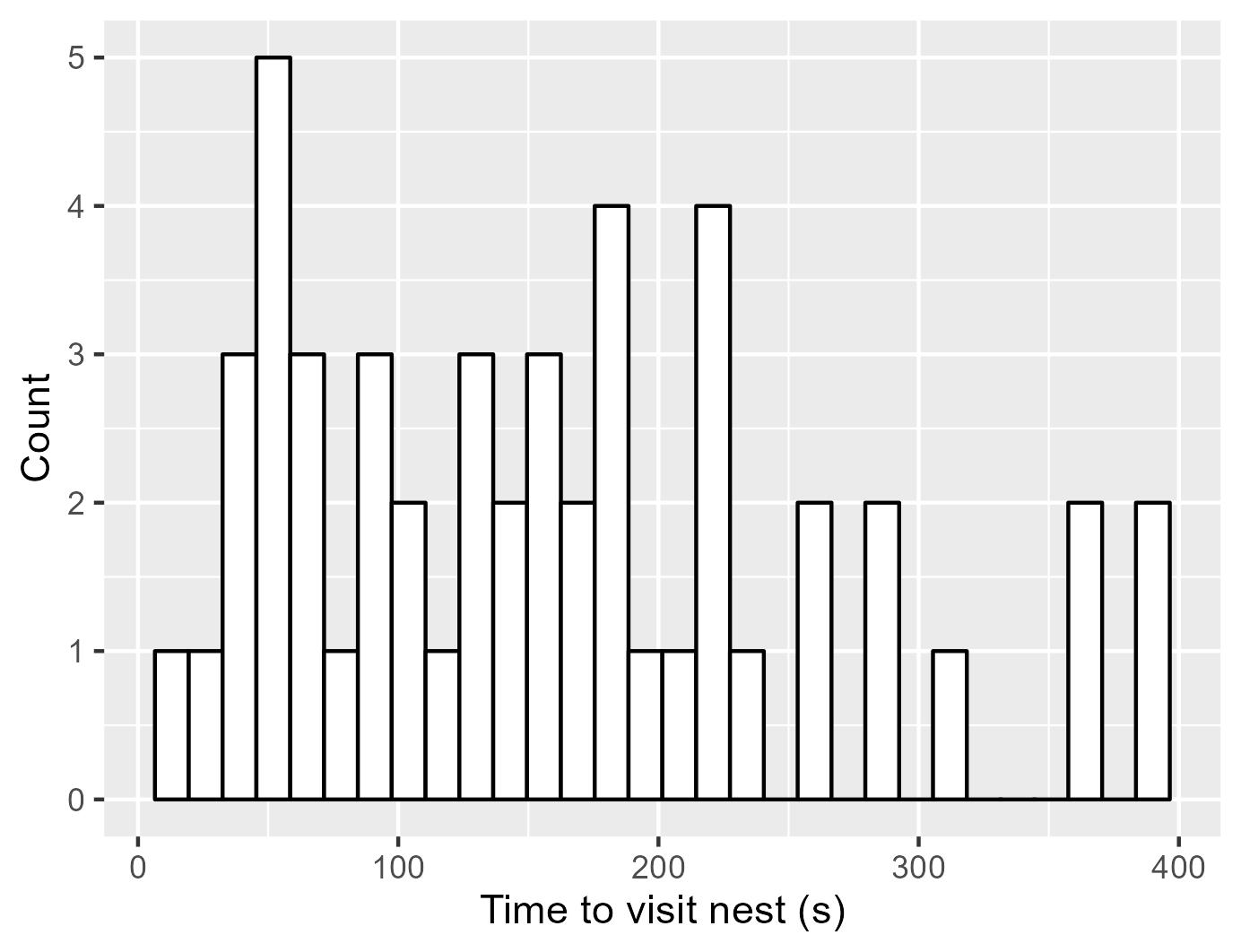


**Figure S1 Time elapsed after common eider flush for a herring gull to visit the nest (n = 50)**


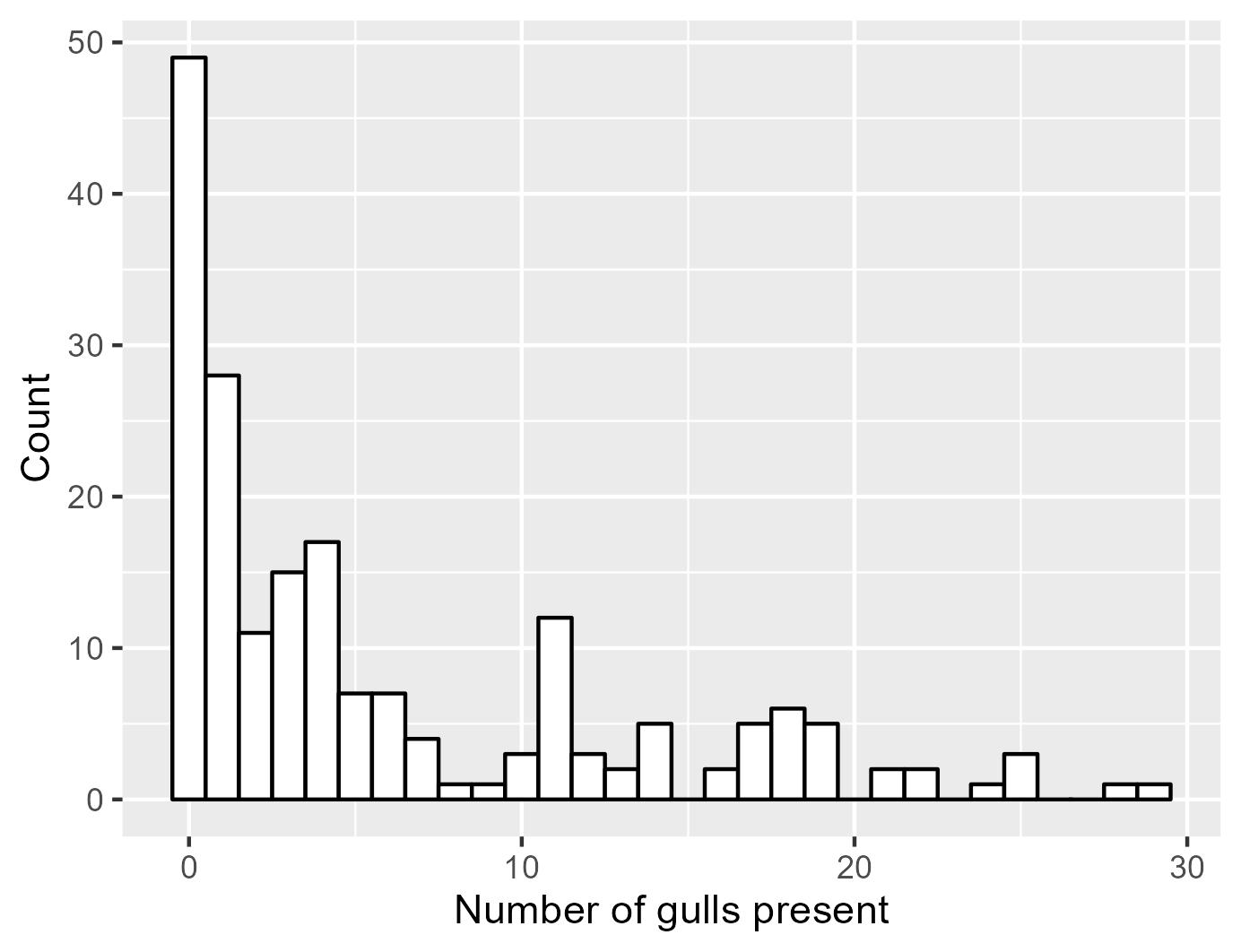


**Figure S2 Number of herring gulls present at time of common eider flush (n = 193)**


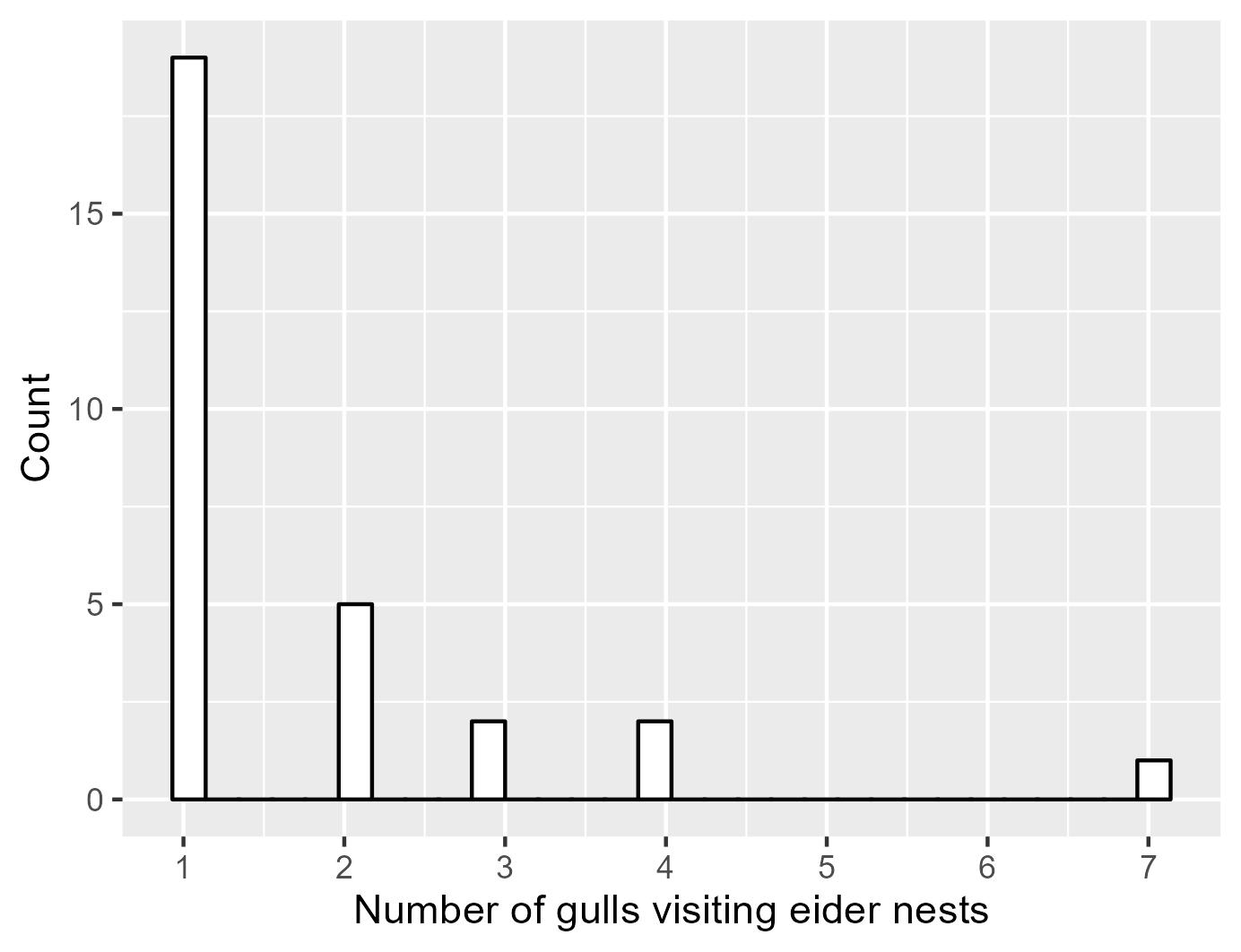


**Figure S3 Number of herring gulls visiting common eider nests (n = 29)**

**Table S1 Residual and Null deviance for the gull visitation and egg consumption model**

| **Model** | **Residual Deviance (DF)** | **Null Deviance (DF)** |
| --- | --- | --- |
| **Gull visitation** | 131.28 (190) | 163.34 (192) |
| **Egg consumption** | 59.202 (47) | 64.104 (49) |
